# Genetic diversity and structure of *Persicaria amphibia* (Polygonaceae) in South Korea using genotyping by sequencing

**DOI:** 10.1007/s13258-024-01571-1

**Published:** 2024-10-19

**Authors:** KyoungSu Choi, Yong Hwang, Jeong-Ki Hong, So Young Park

**Affiliations:** 1 Department of Biology, College of Natural Sciences, Kyungpook National University, Daegu, 41566 Korea; 2 Biological Specimen Conservation Division, Diversity Conservation Research Department, Nakdonggang National Institute of Biological Resources, Sangju, 37242 Korea; 3 Facilities Management Division, Administrative Management Department, Nakdonggang National Institute of Biological Resources, Sangju, 37242 Korea

**Keywords:** *Persicaria amphibia*, GBS, Genetic diversity, Population structure, Phylogenetic relationships

## Abstract

**Background:**

*Persicaria amphibia*, a member of the Polygonaceae family, exists both aquatic and terrestrial forms. It is native to North America, Asia, Europe, and some parts of Africa.

**Objective:**

This study aimed to determine the genetic diversity within and among populations of *P. amphibia* and the distribution characteristics of each population to investigate insights into the genetic structure and conservation of *P. amphibia*.

**Methods:**

In this study, the genetic diversity and structure of 84 *P. amphibia* individuals from 7 populations in South Korea were analyzed using genotyping-by-sequencing (GBS). We used 2,469 single nucleotide polymorphisms (SNPs) to analyze genetic diversity, principal components, structure, and phylogeny.

**Results:**

Our results showed a mean observed heterozygosity and mean expected heterozygosity of 0.34409 and 0.34082, respectively. Genetic diversity analysis indicated that the variation among populations (60.08%) was greater than that within populations (39.92%). Fixation index values, principal components analysis, structure, and phylogeny analyses showed that the population in Gyodongdo, Ganghwa Island was highly different.

**Conclusion:**

These results provide important insights for better understand the population history and genetic structure of *P. amphibia*.

## Introduction

*Persicaria amphibia* is a species of flowering plant originally described as *Polygonum amphibia*, which includes the section *Persicaria* of the genus *Polygonum*. However, due to significant differences in petiole anatomy, life form, and floral characters, the genus *Persicaria* was deemed sufficiently distinct to be classified as its own genus (Decraene and Akeroyd 1988; Ekman and Knutsson 1994). The classification of the genus *Persicaria* has been supported by molecular phylogenetic studies using chloroplast DNA (cpDNA) and nuclear DNA (nrDNA) markers (Kwak et al. 2006; Kim and Donoghue 2008; Kim et al. 2008).

*P. amphibia* (L.) Grey belongs to the genus *Persicaria* (Family Polygonaceae) and is native to North America, Asia, Europe, and parts of Africa (Partridge 2001; Gitsopoulos et al. 2013). It is a rhizomatous perennial with both terrestrial and aquatic forms and is highly variable in morphology (Partridge 2001; Gitsopoulos et al. 2013). The terrestrial form has erect and hairy leaves whereas the aquatic form is hairless, with oblong flat leaves. Both types have similar inflorescence (Partridge 2001; Gitsopoulos et al. 2013). However, Lousley and Kent (1981) suggested that the terrestrial and aquatic forms of *P. amphibia* are different species. Gotsopoulos et al. (2013) investigated the terrestrial and aquatic forms of *P. amphibia* using a combined molecular and morphological approach. Kwak et al. (Kwak et al. 2006) reported 16 taxa of *Persicaria* in Korea and showed that *P. amphibia* was separated from other *Persicaria* species using molecular markers (nrDNA ITS). Similarly, Kim and Donnoghue (2008) reported that *P. amphibia* formed an independent clade using molecular analysis. Recently, Choi et al. (2022) described the chloroplast genome structure and phylogenomics of *P. amphibia*. Overall, studies on the genetic classification of *P. amphibia*, including its terrestrial and aquatic forms, have yielded conflicting results.

*P. amphibia* thrives under sunlight in relatively nutrient-rich water or well-irrigated deep soil (Partridge 2001). Recently, due to human activities and water diversions, aquatic habitats have been fragmented. Consequently, the population of *P. amphibia* in Korea is declining due to limited habitat availability (Yun et al. 2022). However, the habitat conditions and genetic diversity of *P. amphibia* are unclear.

Genetic diversity is widely recognized as an important factor in prioritizing populations for conservation and protection (Moritz 2002; Petit et al. 2008); previous studies evaluated the genetic diversity of populations as part of conservation planning efforts (Lee et al. 2022; Fu et al. 2022; Kang et al. 2023). Genetic diversity has been investigated using various molecular analyses, such as randomly amplified polymorphic DNA (RAPD) (Xu et al. 2003; Ercan et al. 2004), amplified fragment length polymorphism (AFLP) (Simioniuc et al. 2002; Nguyen et al. 2004), inter simple sequence repeat (ISSR) (Ge and Sun 1999; Camacho and Liston 2001) and sequence repeats (SSR) or microsatellites (Ha et al. 2021; Fu et al. 2022). Genotyping by sequencing (GBS) is an application of next-generation sequencing protocols for the discovery and genotyping of single nucleotide polymorphisms (SNPs) in genomes and populations. GBS methods have recently been used for hybridization, genetic diversity, and evolutionary studies in many plant species (Mammadov et al. 2012; Das et al. 2015; Zhao et al. 2022; Dambier et al. 2022).

In the present study, we conducted a genetic diversity analysis of *P. amphibia* using 84 individuals from 7 populations using GBS. The objectives of this study were to determine the genetic diversity within and among populations of *P. amphibia* and the distribution characteristics of each population to provide insights into the genetic structure and conservation of *P. amphibia*.

## Materials and methods

### Sample collection, DNA isolation, library preparation, and NGS sequencing

**Fig. 1:**
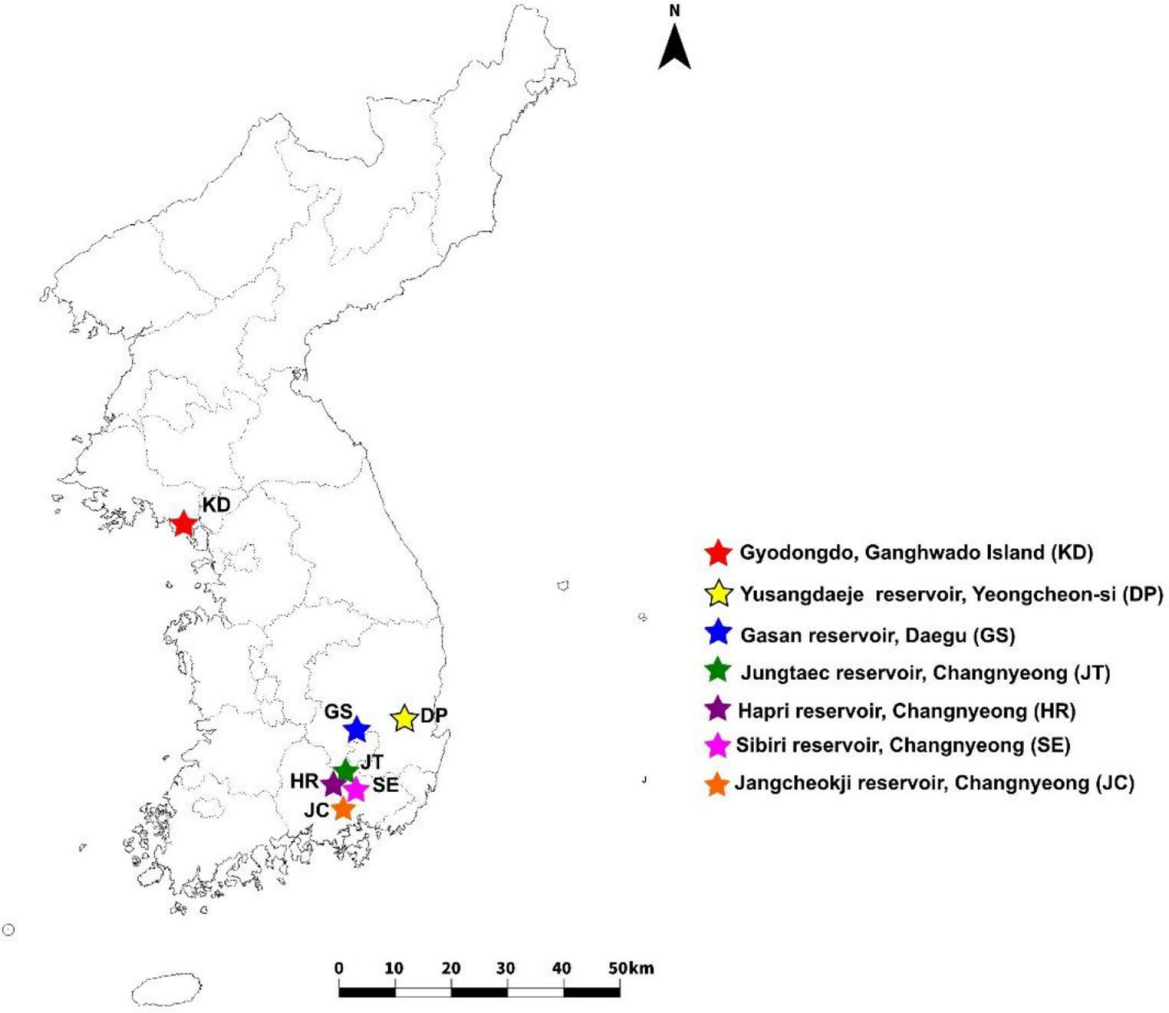
Geographic distribution of the sampling sites of the seven populations of *P. amphibia*

In 2022, we collected leaves from 84 wild individuals from 7 populations, with 10 to 14 individuals in each population (Fig. 1; Table 1). To minimize the number of sample clones, the sampling sites were at least 5 m apart. Leaf tissues were preserved at room temperature in a sealed plastic re-sealable bag with a silica gel desiccant until further use. Total genomic DNA was extracted using the DNeasy Plant Mini Kit (Qiagen Inc., Hilden, Germany), according to the manufacturer’s protocol. The quality of extracted DNA was evaluated and quantified using a Qubit Fluorimeter (Invitrogen, Waltham, MA, USA) and visualized on a 1% agarose gel. The restriction enzymes used for library preparation were *MspI –Pst1* and barcoded with sequence adaptors before pooling into sets of 86 individuals. Each library was amplified by polymerase chain reaction (PCR) and sequenced in a single lane on an Illumina HiSeq X (Illumina Inc., San Diego, CA, USA) using 150 bp paired-end sequencing runs.

**Table 1:** Measures of diversity within 84 *P. amphibia* samples from 7 populations

Pop	*N*	Na	Ne	H_0_	He	F_IS_
KD	14	2	1.64057	0.42187	0.35565	-0.0761
JC	11	2	1.59433	0.41241	0.33574	-0.0652
SE	11	2	1.59932	0.38402	0.34049	-0.0022
HR	10	2	1.64163	0.20868	0.37279	0.49125
JT	12	2	1.62143	0.35412	0.3477	0.07116
DP	12	2	1.65151	0.41154	0.36072	-0.0488
GS	14	2	1.4476	0.21598	0.27266	0.40119
mean			1.59948	0.34409	0.34082	0.11019

*Pop* population group; *N* number of samples; *Na* mean number of alleles; *Ne* mean number of effective alleles; *Ho* observed heterozygosity; *He* expected heterozygosity; *F*_*IS*_, inbreeding coefficient

## Sequencing analyses and SNP identification

The reference genome of *P. sieboldin* was assembled *de novo* to facilitate read-mapping and SNP identification. The draft sequence was pre-processed using DynamicTrim (phred score ≥ 20) and LengthSort (short read length ≥ 25 bp) in the SolexaQA (v.1.13) package (Cox et al. 2010). Clean reads were mapped against the *de novo* assembled reference draft genome using BWA 0.7.17-r1188 (Li and Durbin 2009). The sequence alignment map (SAM) file format was merged and sorted into a binary alignment map (BAM) using SAMtool v. 0.1.16 (Li et al. 2009). The final matrix was generated following the SNPs filtering criteria: SNP loci of biallelic, minor allele frequency > 5%, missing data < 30%, and fixation index *(F*_*ST*_*)* > 0.3.

### Data analysis

For genetic diversity computation, the mean number of alleles (*Na*), mean number of effective alleles (Ne), observed heterozygosity (*Ho*), expected heterozygosity (*He*), and fixation index within populations (or inbreeding coefficient) (*F*_*IS*_) were calculated using ARLEQUIN v.3.5.2.2 (Excoffier and Lischer 2010). Genetic variance was analyzed by computing F-statistics (*F*_*ST*_, *F*_*IS*_, *F*_*IT*_) and using the analysis of molecular variance (AMOVA) following 1000 permutations in ARLEQUIN v.3.5.2.2 (Excoffier and Lischer 2010) using 1,000 permutations. Principal component analysis (PCA) of the 84 samples was based on 2,469 SNPs. The analyses were conducted using the R package SNPRelate.

To evaluate genetic structuring, we assigned individuals to genetic clusters based on Bayesian assignment analysis implemented in STRUCTURE v.2.3.4 (Pritchard et al. 2000). Population clustering with the admixture and correlated allele frequency models was selected with 10,000 burn-in steps followed by 100,000 runs of Markov chain Monte Carlo (MCMC). Ten independent STRUCTURE runs were repeated for each K from 1 to 10.

Evolutionary history was inferred using the neighbor-joining method with 1000 replications. The optimal tree with a sum of branch lengths = 8.78408112 is shown. The evolutionary distances were computed using the Maximum Composite Likelihood method and are expressed in units of the number of base substitutions per site. The analysis involved 86 nucleotide sequences. All ambiguous positions were removed from each pair of sequences. There were 2,469 positions in the final dataset. Evolutionary analyses were conducted using MEGA6 (Tamura et al. 2013).

## Results

### SNP analysis

A total of 794,289,086 bp raw reads were obtained from the 86 *P. amphibia* samples. After filtering out low-quality data, an average of 8,690,189 bp was obtained. The filtered sequences were compared to the *P. maackiana* reference genome. The average of mapped reads of the 84 *P. amphibia* samples was 78.83%. In the GBS analysis, a total of 409,201 called and unfiltered SNPs were detected as raw SNP markers. Of these, 10,514 filtered SNPs (minor allele frequency < 5%, missing data < 30%) were obtained, and the final filtering step detected 2,469 SNPs (*F*_*ST*_ > 0.3) from 84 genotyped individuals across 7 populations of *P. amphibia*.

### Genetic diversity

The mean numbers of alleles (*Na*) and effective alleles (*Ne*) were 2 and 1.59948, respectively. The observed heterozygosity (*Ho*) and expected heterozygosity (*He*) ranged from 0.20868 (HR) to 0.42187 (KD) and 0.27266 (GS) to 0.37279 (HR), respectively (Table 1). The inbreeding coefficient (*F*_*IS*_) of the 7 populations of *P. amphibia* ranged from − 0.0022 (SE) to 0.49125 (HR). The HR population was higher than that of other populations, and the F_IS_ of the KD, JC, SE, and DP populations was negative.

## Geographic distance and genetic differentiation

**Fig. 2:**
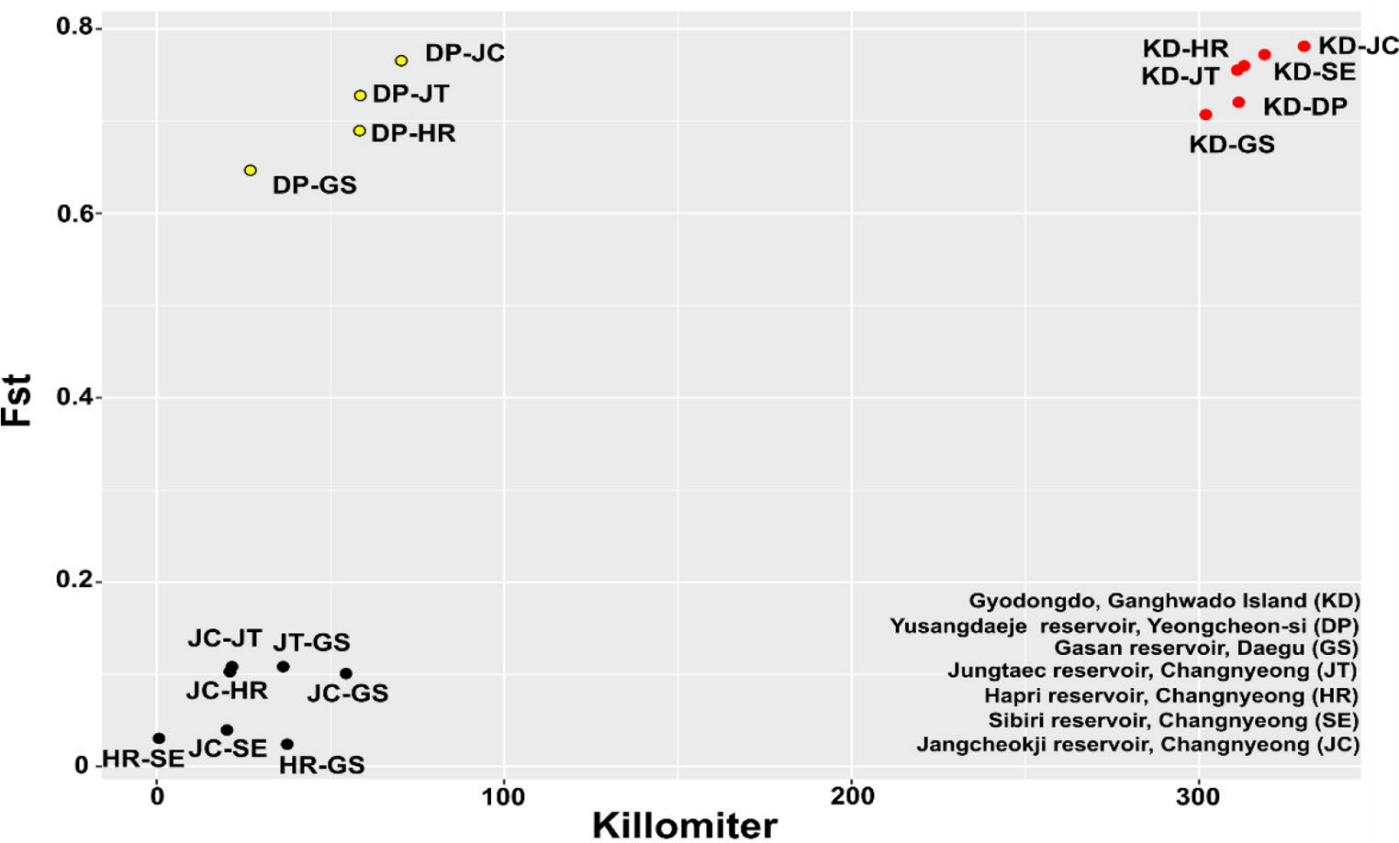
Geographic distance (km) and *F*_*ST*_ among seven populations

We performed AMOVA to determine the genetic differences between and within the populations. The results showed that 60.08% of variation occurred among populations and 39.92% (-2.695% + 42.61%) occurred within a population (Table 2). The pairwise *F*_*ST*_ between populations ranged from 0.02389 (HR-GS) to 0.78137 (KD-JC), with a mean of 0.4611. The KD population showed high genetic differentiation from other populations (Fig. 2). The KD and DP populations had significantly higher *F*_*ST*_ than other populations (DP, GS, JT, HR, SE, and JC). Moreover, there were close genetic relationships among the four populations JT, HR, SE, and GS.

**Table 2:** Analysis of molecular variance (AMOVA) of *P. amphibia* based on 2,469 SNPs

	Df	Sum of squared	Variance component	Variation (%)	F-statistics
Among population	**6**	**16817.103**	0.42187	60.08	F_ST_ = 0.60080
Among individuals (within populations)	77	5445.004	0.41241	-2.69	F_IS_ = -0.06728
Within individuals	84	6797.000	0.38402	42.61	F_IT_ = 0.57394
Total	167	29059.107	0.34409	100	

*F_*ST*_: estimated differentiation among groups, *F*_*IS*_: estimated differentiation within groups, *F*_*IT*_: estimated differentiation within groups

## PCA and population structure analysis

**Fig. 3:**
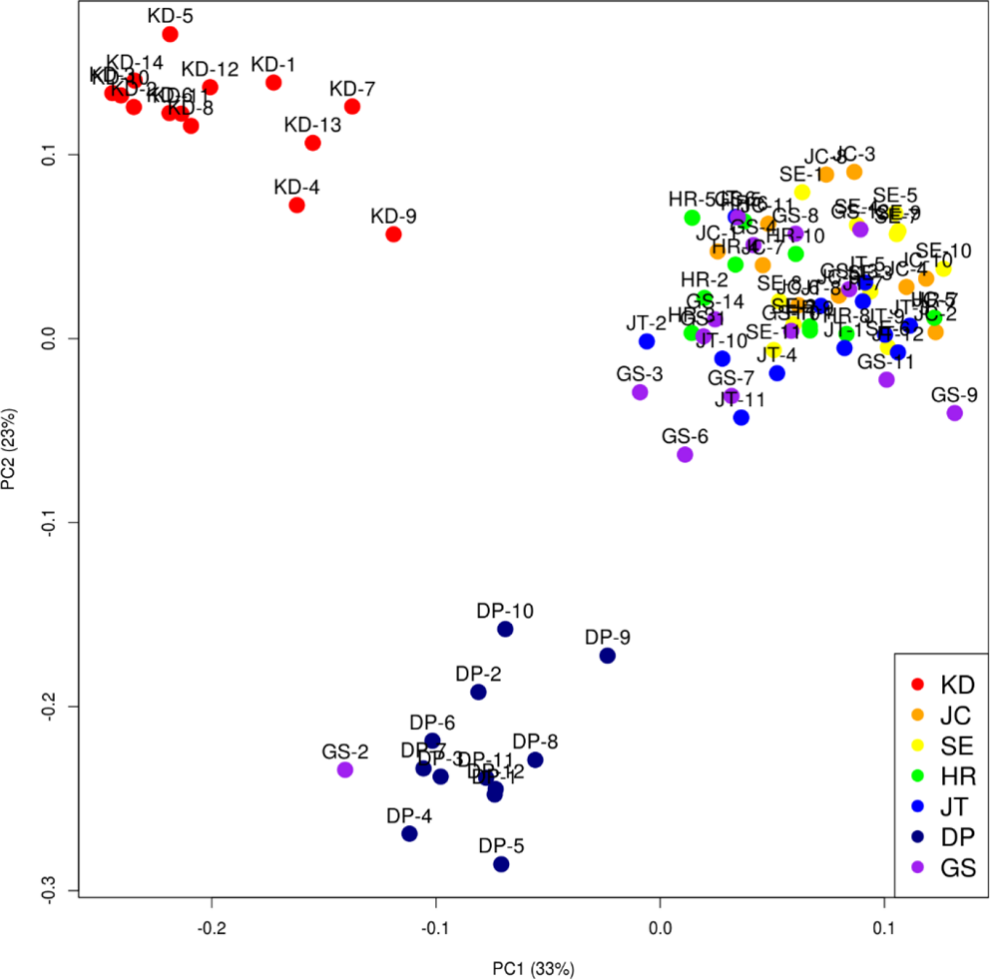
Results of principal component analysis (PCA) of genetic distances for 84 genotyped individuals of *P. amphibia*

**Fig. 4:**
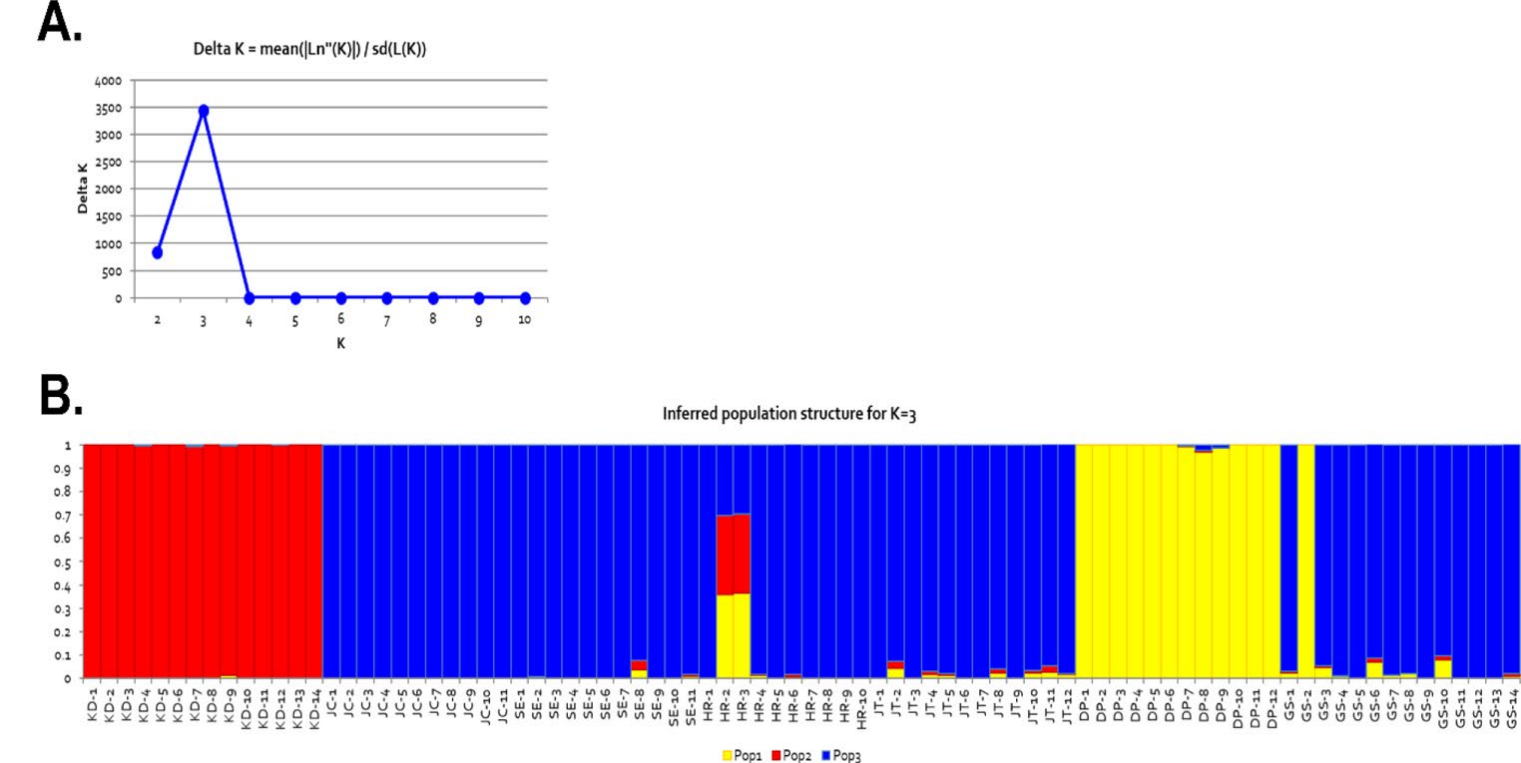
Population structure of the 84 P. amphibia individuals from 7 populations samples in this study. (**A**) The plot of the ∆K value with the number of subpopulations (K) from 2 to 10. The peak value of ∆K was at K = 3. (**B**) Populations structure of the 84 *P. amphibia* samples collected from 7 populations

We used PCA and STRUCTURE to study the genetic relationships among the 84 *P. amphibia* individuals from 7 populations in South Korea. A plot of the first two principal components (PCA) indicated that sufficient variation was captured within PC1 (33%) and PC2 (23%). The PCA results showed that the individuals in the KD and DP populations clustered together in each population and separated the individuals in other populations, except for one individual in the GS-2 group (Fig. 3). In the population structure of the 84 individuals of *P. amphibia* from 7 populations, the highest values of ∆K were *K* = 3 (Fig. 4). PCA revealed three main clusters corresponding to the three ancestral groups identified using STRUCTURE. The red and yellow ancestral groups were mostly composed of KD and DP (including GS-2). The blue group included five groups JC, SE, HR, JT, and GS. However, two individuals (HR-2 and HR3) in the HR were mixed with the three ancestor populations (Fig. 4).

### Phylogenetic analysis

**Fig. 5:**
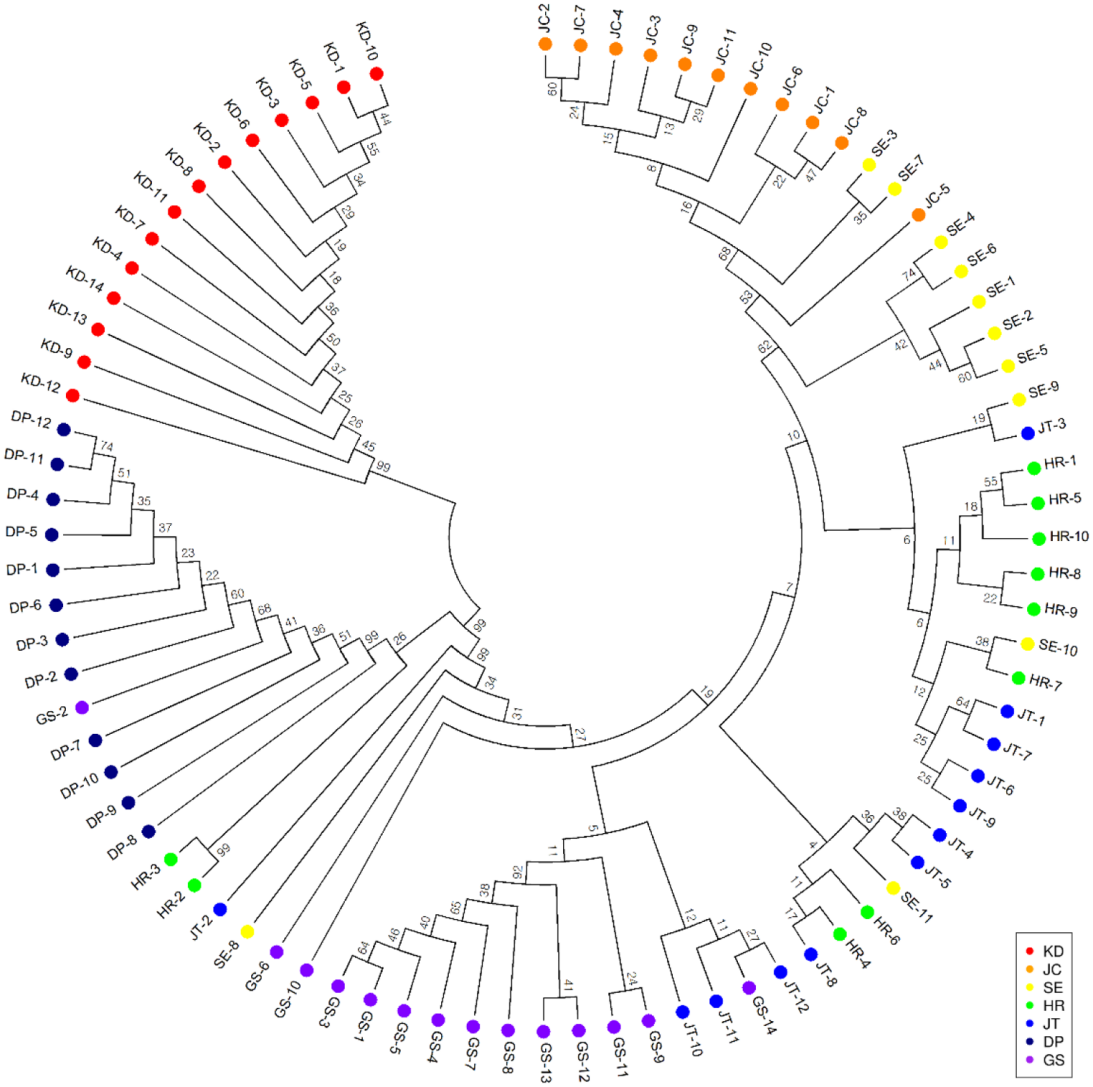
The neighbor-joining tree of the 84 *P*. *amphibia* individuals. Node values correspond to bootstrap values

We performed a phylogenetic analysis using the MEGA6 program based on 2,469 SNPs to establish a neighbor-joining tree divided into 2 main groups (Fig. 5). The KD population was separated from the other populations, the six populations were clustered, and there were close genetic relationships.

## Discussion

Genetic diversity is an important factor in environmental change and plant adaptation. Narrowly distributed and endemic plants show lower genetic diversity than plants in wider distribution areas (Hamrick and Godt 1990). Low diversity has been attributed to inbreeding and drifting in small populations (DeJoode and Wendel 1992). In this study, we genotyped 84 *P. amphibia* individuals collected from 7 populations (Fig. 1; Table 1). The observed and expected heterozygosity of *P. amphibia* populations were both less than 0.5, particularly the expected heterozygosity of the GS population (0.27266) was significantly lower than that of other populations. Inbreeding leads to an increase in homozygosity, reduced performance of production traits, and reduced fitness (Ouborg et al. 2010). The signs of within-population inbreeding were identified at three sites (HR, JT, and GS populations), which is likely because the three habitats (HR, JT, and GS populations) of *P. amphibia* were smaller than those of the other populations.

*P. amphibia* have lower genetic diversity than other endangered Polygonaceae species, such as *Rheum tanguticum* (*He* = 0.515) (Chen et al. 2009). In the present study, the mean *He* value (0.34082) of *P. amphibia* was similar to those of endangered plants in Korea, such as *Pelatantheria scolopendrifolia* (*H e* = 0.356) (Yun et al. 2020), *Saussurea polylepis* (*He* = 0.43) (Yun and Kim 2021) and *Abeliophyllum distichum* (*He* = 0.319) (Lee et al. 2022). Moreover, genetic variation among *P. amphibia* populations (60.08%) was higher than within populations (39.92%). Hamrick and Godt (Hamrick and Godt 1990) have revealed that selfing species have over 50% of the variation among populations. *P. amphibia* is self-incompatible species (Partridge 2001), which explains our results of the high genetic variation (over 60%) among populations of *P. amphibia*.

The fixation index *F*_*ST*_ has been widely used as a measure of population structure; Wright (1984) has suggested that an F_ST_ in the range of 0 to 0.05 indicates no genetic differentiation among populations whereas that in the range of 0.05–0.15 indicates moderate differentiation (Slatkin 1985; Shu et al. 2021). In the present study, the F_ST_ values between four populations (HR, GS, SE, and JC or HR-GS, HR-SE, and JC-SE) were smaller than 0.05 or close to 1 (JC-GS, JC-HR, and JT-GS) whereas those between KD and DP populations were considerably high, indicating that these two populations are highly differentiated. This phenomenon is considered to be related to the pollinator (Gamba and Muchhala 2023); for *P. amphibia*, insects are known pollinators (Harms and Grodowitz 2009). In addition, the distance between the KD population and other populations was over 300 km, and that between the DP population and other populations was over 50 km, which may explain the considerably high *F*_*ST*_ values of the two regions.

In the present study, the spatial clustering pattern suggests three ancestral populations. At *K* = 3, five populations (JC, SE, HR, JT, and GS) were assigned to a single cluster and two populations (KD and DP) were clearly separated. For the KD population, we suggest that it represents a different cluster because it is located on an Island 500 km away from other populations. Moreover, although the DP population was not geographically far (in terms of distance), it was not connected to other populations in the same river. Notably, both phylogenetic and principal component analyses showed results similar to those of the genetic analysis. The neighbor-joining tree also revealed that the KD population of *P. amphibia* formed one clade whereas the other populations (DP, JC, SE, HR, JT, and GS) formed one clade. Five populations (JC, SE, HR, JT, and GS) were closely clustered into one group, and the DP population sister group clustered with five populations (JC, SE, HR, JT, and GS). This result may be because these five populations (JC, SE, HR, JT, and GS) were closely located, indicating that their genetic relationships were also close.

### Conclusions

GBS is an NGS protocol used for the discovery and genotyping of SNPs in genomes and populations. GBS methods have been applied in a wide range of studies, including genetic diversity, phylogenic classification, and hybridization analysis. This study detected 2,469 SNPs to estimate the genetic diversity and population structure of 84 *P. amphibia* individuals from 7 populations. We found that the seven populations were clustered into three groups. In particular, the F_ST_ values and structural analyses of the KD population were highly different among the seven populations. These phenomena can be explained by their geographic location. This is the first study of the genetic diversity of *P. amphibia*, providing important insights into the population history, genetic structure, and population conservation of *P. amphibia*.

## Data Availability

All data generated or analyzed during this study ar included in this published article.
